# Cystoscopy-Guided Laparoscopic Excision of Prostatic Utricle: Report of a Case

**DOI:** 10.1055/s-0040-1705155

**Published:** 2020-04-28

**Authors:** Ozlem Boybeyi-Turer, Huseyin Demirbilek, Tutku Soyer

**Affiliations:** 1 Department of Pediatric Surgery, Hacettepe University Faculty of Medicine, Ankara, Turkey; 2 Department of Pediatric Endocrinology, Hacettepe University Faculty of Medicine, Ankara, Turkey

**Keywords:** prostatic utricle, cystoscopy, laparoscopy

## Abstract

Prostatic utricle (PU) is incomplete regression of Müllerian duct and may cause recurrent urinary tract infections (UTIs), stone formation, postvoid dribbling, and recurrent epididymitis. Although surgical excision is recommended to avoid complications, surgical access to PU has been challenging. Cystoscopy-guided laparoscopic management of PU in a 3-year-old boy is reported to discuss use of other endoscopic aids in the surgical treatment of PU. He was admitted with disordered sexual development with karyotype of 47,XYY/46,XY and has been experiencing recurrent UTIs. Voiding cystourethrogram (VCU) demonstrated large PU (IKOMA II). Cystoscopy was performed confirming PU and the cystoscope was left in situ to aid laparoscopic exploration after bladder was emptied. A 5-mm umbilical port and two 5-mm ports in both lower quadrants were inserted. The peritoneum was dissected behind bladder. The cystoscope in PU was used as guidance in identification and dissection of PU. The vas deferens was identified and could be secured. The neck of PU was ligated with surgiloop. PU was retrieved from umbilical port. Postoperative VCU revealed normal posterior urethra. He has been free of UTIs for the last 6 months. Laparoscopy is safe and feasible alternative in surgical management of PU, by providing good visual exposure, easy dissection in deep pelvis, and improved cosmesis. The cystoscopic guidance is an important aid in identification and dissection of PU.

## Introduction

A prostatic utricle (PU) is an enlarged diverticulum that communicates with the posterior urethra. PUs occur due to incomplete regression of the Müllerian ducts and are commonly associated with hypospadias, cryptorchidism, and intersex disorders. 1 2 The precise incidence of PU is unknown; however, it is observed in 14% of proximal hypospadias patients and 57% of perineal hypospadias patients. 3 The age of presentation varies between 2 months and 75 years, but most of the cases present during childhood. 3 Although most PUs are asymptomatic, ∼30% of PUs cause recurrent urinary tract infections (UTIs), stone formation, postvoid dribbling, and recurrent epididymitis. 1 2 3 In addition to clinical symptoms, voiding cystourethrography (VCU) is used for the diagnosis and classification of PU. PU is classified as follows: grade 0—confined to the verumontanum; grade 1—does not reach the bladder neck; grade 2—the dome extends over the bladder neck; and grade 3—the opening of the PU is distal to the external sphincter. 4

Surgical excision is the treatment of choice in symptomatic cases; however, gaining surgical access to PUs is challenging. Several open surgical techniques have been described to date, including transperitoneal, perineal, posterior sagittal, and transvesical approaches 2 5 ; however, most of these techniques are associated with the risk of damaging the pelvic structures, poor exposure, incomplete excision of the PU, and prolonged hospitalization. 3 6 Laparoscopic management of PUs is becoming more common as the surgical treatment of choice following Yeung et al's 3 report that laparoscopic excision of PUs facilitates good exposure and a low complication rate. Herein we report the use of cystoscopy-guided laparoscopic excision of a PU in a male pediatric patient.

## Case Report

**Fig. 1:**
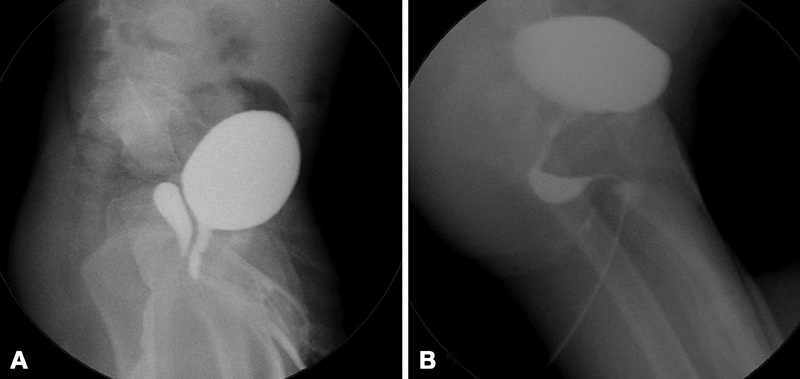
( **A** ) The retrograde urethrography demonstrates large prostatic utricle extending over bladderneck (IKOMA Grade 2), and ( **B** ) the postoperative urethrography revealing normal posterior urethra.

A 3-year-old boy presented with disordered sexual development and the 47,XYY/46,XY karyotype. He had penoscrotal hypospadias, bifid scrotum, and asymmetrical gonads. He had been experiencing UTIs (10 ^5^ colony-forming unit [CFU] of *E. coli* ) more than six times annually. VCU and retrograde urethrography showed a grade 2 PU ( Fig. 1A ) and because of the persistent UTIs surgical excision of the PU was scheduled.

Under general anesthesia, cystoscopy was performed to confirm the patient's PU. The cystoscope (Karlz-Storz 9F pediatric cystoscope) was inserted into the 3-cm long utricle and left in situ to aid laparoscopic exploration. The bladder was emptied via the working channel of the cystoscope before the cystoscope was left in the utricle. A 5-mm umbilical port was inserted via the Hassan technique and a pneumoperitoneum was created. Then, two additional 5-mm ports were inserted in the right and left mid-abdomen under direct vision. The peritoneal reflection was incised just behind the bladder using electrocautery.

**Fig. 2:**
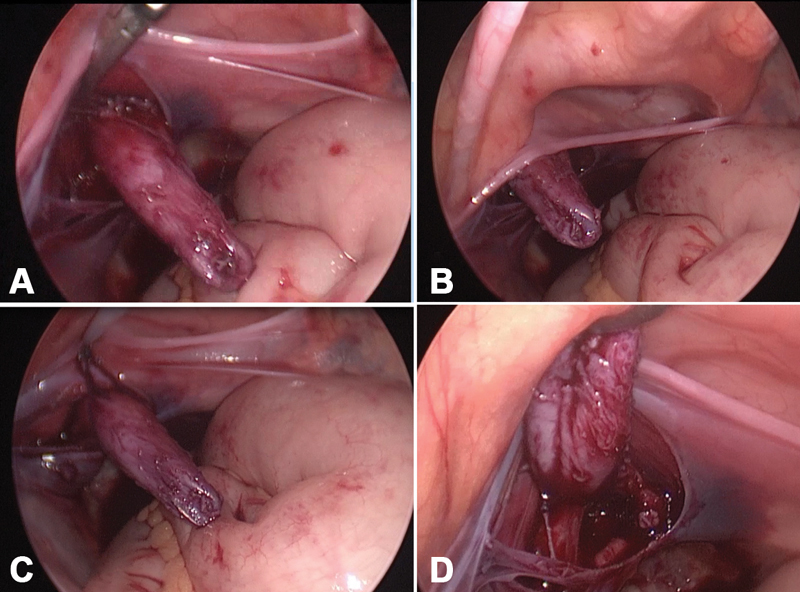
The operative view showing ( **A** ) the retracted cystoscope within the prostatic utricle (PU), ( **B** ) PU below peritoneal reflection, ( **C** ) ligation of endoloop, and ( **D** ) PU after ligation and withdrawal of cystoscope.

The cystoscope was used to guide identification and dissection of the PU ( Fig. 2 ). The indwelling cystoscope further assisted the dissection by lifting and providing countertraction of the PU. The bilateral vas deferens was identified and successfully secured. The PU was then completely mobilized and divided from its connection to the urethra after using a single Ethicon surgiloop to ligate the neck of the PU. The excised PU was removed via the umbilical port. Postoperative urethrography showed a normal posterior urethra ( Fig. 1B ). During 6 months of postsurgical follow-up, the patient did not have any UTI. The parents of the patient provided written informed consent to use the patient's clinical data for scientific purposes.

## Discussion

Embryologically, PUs resemble the caudal end of the Müllerian canal, which regresses in males. 1 2 The anatomical location of PUs at the posterior urethra in between the ejaculatory ducts makes both its diagnosis and surgical management challenging. Most PU cases are asymptomatic, but ∼33% of cases present with recurrent UTIs, stone formation, postvoid dribbling, and recurrent epididymitis. 1 2 3 Additionally, neoplastic degeneration occurs in ∼3% of adolescent and adult PU cases. 3 7 8 For instance, Gualco et al 7 reported a 16-year-old male that developed clear cell adenocarcinoma and Zhang et al 8 reported a 39-year-old male that developed squamous cell carcinoma from an enlarged PU; therefore, clinical suspicion is the first diagnostic step. Although pelvic ultrasonography can identify a cystic mass behind the bladder, VCU is the examination of choice for a precise PU diagnosis. Cystoscopy is performed in almost all cases to confirm the diagnosis and to determine the size of the PU. 1 5 Meisheri et al 5 recommends retrograde urethrography and cystoscopy in all proximal hypospadias cases and proposed a therapeutic algorithm based on the grade of the utricle. The presented patient had hypospadias associated with a PU and presented with resistant recurrent UTIs. After confirming the diagnosis of PU via VCU, he was scheduled to undergo excision of the large PU due to recurrent UTIs.

Large and symptomatic PUs should be excised surgically—not only for relief of the symptoms, but also to obtain evidence of malignancy arising from Müllerian duct remnants. 7 8 9 In addition to the difficulty dissecting PUs deep in the pelvis, preservation of fertility is another challenge in the surgical management of PUs because of their anatomical proximity to the spermatic cords; therefore, several surgical approaches for excision of PUs have been described, 3 although to date, there is no standard approach according PU size.

Open surgical techniques are associated with the risk of incomplete PU excision, damage to vital pelvic structures, and poor exposure. 3 6 The most common open techniques are transurethral, transvesical, transperitoneal, perineal, and posterior sagittal—with or without the transrectal approach. 2 3 5 6 Although the transperitoneal approach facilitates intrapelvic organ exploration, dissection deep in the pelvis can be difficult. 5 The perineal approach is associated with an increased risk of damage to the pudendal nerve, external sphincter, and rectum. 5 To improve exposure and ease of dissection, the transvesical approach is recommended 5 6 ; however, the transvesical approach can interfere with trigonal function. 5 6 It has been suggested that the best exposure is achieved via the posterior sagittal approach. 3 5 Moreover, preoperative bowel preparation and the necessity of incising the rectum when using the transrectal approach are considered disadvantages of the method. 3 4 5

Because of the difficulties associated with open surgical approaches, laparoscopic management was proposed in 2001 by Yeung et al. 3 Subsequently, several case reports have described some technical differences, such as cystoscopic guidance, bladder stitch, and use of endoclip or endoloop for ligation of PUs. 1 2 10 11 Laparoscopic excision of PUs provides good exposure, improved wound cosmesis, complete excision, and a lower complication rate. 2 3 6 Jia et al 6 reported significantly shorter hospitalization, lower estimated blood loss, and shorter surgical duration using the laparoscopic approach, as compared with open procedures. 6 They also noted a residual utricle stump in three cases following open excision of a PU, versus no such instances following the laparoscopic approach. 6 Additionally, it is not necessary to incise the bladder or rectum when using the laparoscopic approach. The use of a cystoscope placed in the PU improves identification of the utricle and provides exposure that is superior to that of open techniques. The most difficult part of PU excision is closure of the urethral opening with laparoscopic suturing techniques 3 ; therefore, an endoclip or endoscopic surgiloop can be used safely to ligate and close the connection to the urethra before excision of the PU. 6 12 In the presented case, the connection to the urethra was ligated using a surgiloop. In addition, emptying the bladder and suspension of the bladder have been recommended to improve exposure 6 ; however, in the present case the bladder was emptied, but the bladder was not suspended because the exposure was sufficient for identifying the PU. We think that the presented case shows that clinicians should consider use of the laparoscopic approach, so as to avoid unnecessary suprapubic bladder puncture and/or bladder stitching during PU excision. Moreover, identification of the spermatic cords with the magnification of a laparoscope facilitates safer surgery.

## Conclusion

In conclusion, laparoscopic excision of PUs should be considered a feasible alternative to open techniques. It provides good visual exposure, easy dissection deep in the pelvis, and improved wound cosmesis. Cystoscopic guidance is an important aid for identifying and dissecting PUs. Ligation of the PU neck using a surgiloop is also a practical way to close the urethral opening in selected cases. Emptying the bladder during cystoscopy may be sufficient for optimal visualization in selected cases.

## Notes

New Insights & the Importance for the Pediatric Surgeon

- Laparoscopy may be safe and feasible alternative in surgical management of prostatic utricle.
- The cystoscopic guidance is an important aid in identification and dissection of prostatic utricle.

## References

[1] Liu B He D Zhang D Liu X Lin T Wei G Prostatic utricles without external genital anomalies in children: our experience, literature review, and pooling analysis BMC Urol 2019 19 01 21 30943976 10.1186/s12894-019-0450-z PMC6446365

[2] Mostafa I A Woodward M N Shalaby M S Cystoscopic-assisted laparoscopic excision of prostatic utricle J Pediatr Urol 2018 14 01 77 78 29137943 10.1016/j.jpurol.2017.09.024

[3] Yeung C K Sihoe J D Tam Y H Lee K H Laparoscopic excision of prostatic utricles in children BJU Int 2001 87 06 505 508 11298044 10.1046/j.1464-410x.2001.00132.x

[4] Ikoma F Shima H Yabumoto H Classification of enlarged prostatic utricle in patients with hypospadias Br J Urol 1985 57 03 334 337 4005502 10.1111/j.1464-410x.1985.tb06356.x

[5] Meisheri I V Motiwale S S Sawant V V Surgical management of enlarged prostatic utricle Pediatr Surg Int 2000 16 03 199 203 10786981 10.1007/s003830050722

[6] Jia W Liu G C Zhang L Y Comparison of laparoscopic excision versus open transvesical excision for symptomatic prostatic utricle in children J Pediatr Surg 2016 51 10 1597 1601 27339083 10.1016/j.jpedsurg.2016.06.004

[7] Gualco G Ortega V Ardao G Cravioto F Clear cell adenocarcinoma of the prostatic utricle in an adolescent Ann Diagn Pathol 2005 9 03 153 156 15944958 10.1016/j.anndiagpath.2005.02.006

[8] Zhang C Li X He Z Xiao Y Li S Zhou L Squamous cell carcinoma of the enlarged prostatic utricle in an adult Urology 2012 79 02 e23 e24 21820701 10.1016/j.urology.2011.06.017

[9] Lima M Maffi M Di Salvo N Robotic removal of Müllerian duct remnants in pediatric patients: our experience and a review of the literaturePediatr Med Chir2018 40(01). Doi: 10.4081/pmc.2018.182 10.4081/pmc.2018.182 29871477

[10] Nguyen A Arora H Reese J Kaouk J Rhee A Robot-assisted laparoscopic excision of prostatic utricle in a 3-year old J Pediatr Urol 2018 14 04 343 344 30396603 10.1016/j.jpurol.2018.07.015

[11] Hester A G Kogan S J The prostatic utricle: an under-recognized condition resulting in significant morbidity in boys with both hypospadias and normal external genitalia J Pediatr Urol 2017 13 05 4920 4.92E7 10.1016/j.jpurol.2017.01.019 28319024

[12] Willetts I E Roberts J P MacKinnon A E Laparoscopic excision of a prostatic utricle in a child Pediatr Surg Int 2003 19 07 557 558 13680292 10.1007/s00383-003-0993-6

